# Engineering of a Biomimetic Interface between a Native Dental Tissue and Restorative Composite and Its Study Using Synchrotron FTIR Microscopic Mapping

**DOI:** 10.3390/ijms22126510

**Published:** 2021-06-17

**Authors:** Pavel Seredin, Dmitry Goloshchapov, Yuri Ippolitov, Jitraporn Vongsvivut

**Affiliations:** 1 Solid State Physics and Nanostructures Department, Voronezh State University, University sq.1, 394018 Voronezh, Russia; goloshchapovdl@gmail.com; 2 Scientific and Educational Center “Nanomaterials and Nanotechnologies”, Ural Federal University named after the first President of Russia B. N. Yeltsin, Mir av., 620002 Yekaterinburg, Russia; 3 Department of Pediatric Dentistry with Orthodontia, Voronezh State Medical University, Studentcheskaya st. 11, 394006 Voronezh, Russia; dsvgma@mail.ru; 4 ANSTO—Australian Synchrotron, 800 Blackburn Road, Clayton, VIC 3168, Australia; jitrapov@ansto.gov.au

**Keywords:** biomimetic, hybrid biointerface, dental tissue, restoration, nanodentistry

## Abstract

The aim of this work is to develop a biomimetic interface between the natural tooth tissue and the restorative composite and to study it on the basis of synchrotron micro-FTIR mapping and multidimensional processing of the spectral data array. Using hierarchical cluster analysis of 3D FTIR data revealed marked improvements in the formation of the dentine/adhesive/dental hybrid interface using a biomimetic approach. The use of a biomimetic strategy (application of an amino acid–modified primer, alkaline calcium and a nano-c-HAp–modified adhesive) allowed the formation of a matrix that can be structurally integrated with natural dentine and dental composite. The biomimetic hybrid layer was characterised by homogeneous chemical composition and a higher degree of conversion of the adhesive during polymerisation, which should provide optimal integration of the dental composite with the dentine.

## 1. Introduction

Long-term, high-quality restoration of lost dental tissue requires appropriate organisation of the dentine–adhesive–dental material interface [1,2,3,4]. As high morphological organisation of the prepared dentine surface determines the structure of the dentine/composite interface, it must be monitored and controlled [2,3,5,6,7].

There are currently a variety of naturally derived bonding systems used to create a stable bond between restorative material and dental hard tissue [2,3,8]. In general, micro-mechanical bonding/adhesion occurs between the restorative material and the etched apatite prisms via formation of a hybrid layer within the demineralised collagen network [3,8,9,10]. The interface quality and affinity of the hybrid layer for the restorative material, which is determined by complex molecular interactions between the bond and the natural dental hard tissue, influence the longevity of the dental restoration [11,12]. Existing defects in the dentine/dental material interface prevent sustainable enamel/dentine restoration [4,13,14]. Restorative composites have disparate functional properties (e.g., mechanical strength, elasticity) from natural dental hard tissue, which, coupled with poor affinity for and integration into the dental tissue, can result in microleakage [15], secondary caries [1] and failure of the resin-dentine interface [4,13].

Modern hybrid layer formation strategies rely on nanotechnology [11,12,16]. Engineering an optimal hybrid biointerface requires materials having: (1) maximum affinity for natural enamel and dentine apatite and (2) similar morphological features and chemical composition as the natural amino acid matrix [17,18,19]. We and others have confirmed that a biomimetic hybrid layer comprised of nanocrystalline carbonate-substituted hydroxyapatite (nano-c-HAp) and polar amino acids found in the enamel matrix can form a qualitative biointerface between synthetic material and natural hard tissue [17,20,21,22,23,24]. This system improves integration of the synthetic materials [17,20,25], making the transitional hybrid layer less problematic for restorative dentistry [11]. Alkaline media has also proven beneficial in biomimetic systems, as it mimics the properties of the enamel matrix or dentine to support stable bond formation at the biointerface [12,21].

Fourier transform infrared (FTIR) microscopy can be used to elucidate the mechanisms for integration (molecular bonding) of the hybrid layer with dental tissue and restorative composites and assess the chemical composition, structure, thickness and quality of the hybrid biointerface [7,22,23]. FTIR maps of functional groups (FTIR chemometrics) can reveal the chemical composition throughout a material/tissue and the spatial variation of chemical properties within a small volume of biomaterial [7,24,26]. Unfortunately, one-dimensional mapping cannot always resolve the subtle spectral differences between samples that report on molecular interactions. Implementing multivariate statistical analysis on an array of spectral data (cluster analysis), along with visualisation, could be an effective tool for biochemical fingerprinting and assessing the integration of the biomimetic interface between dental tissue and composites [7,27]. However, there are few reports on the use of FTIR chemometrics coupled with multivariate statistical analysis to study the molecular chemical properties of composite material/natural tissue interfaces.

Thus, the aim of this work is to develop of a biomimetic interface between the natural tooth tissue and the restorative composite and to study it on the basis of synchrotron micro-FTIR mapping and multidimensional processing of the spectral data array.

## 2. Results

### 2.1. FTIR Chemical Imaging

**Figure 1:**
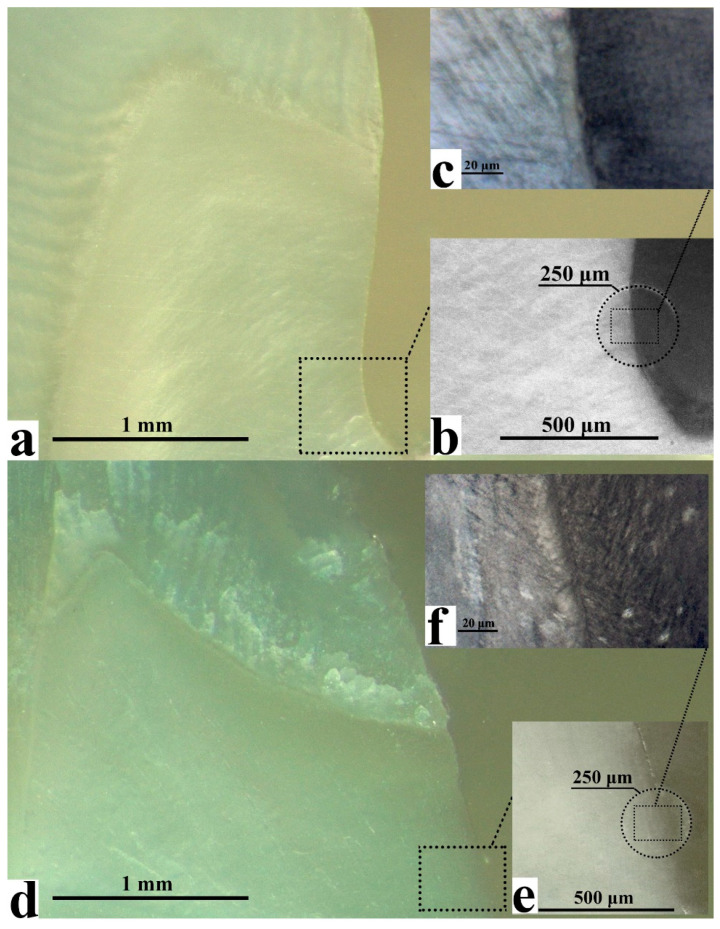
Representative optical images of interface areas for type I (**a**–**c**) and type II- biomimetic (**d**–**f**) samples at different magnifications.

The healthy dentine/adhesive/dental material interface of all type I and type II specimens (*n* = 10) were examined using high resolution (~1 μm) FTIR microspectroscopy. The analysed areas were selected using an optical microscope within the measuring circuit. Interface areas of 30 × 50 μm^2^ showing no mechanical (e.g., polishing) or other defects were selected for detailed analysis. The images of both type I and type II specimens clearly show two areas: healthy dentine and adhesive/dental material (Figure 1). As optical imaging does not provide greater resolution, FTIR mapping was employed to investigate the specimens in greater detail.

**Figure 2:**
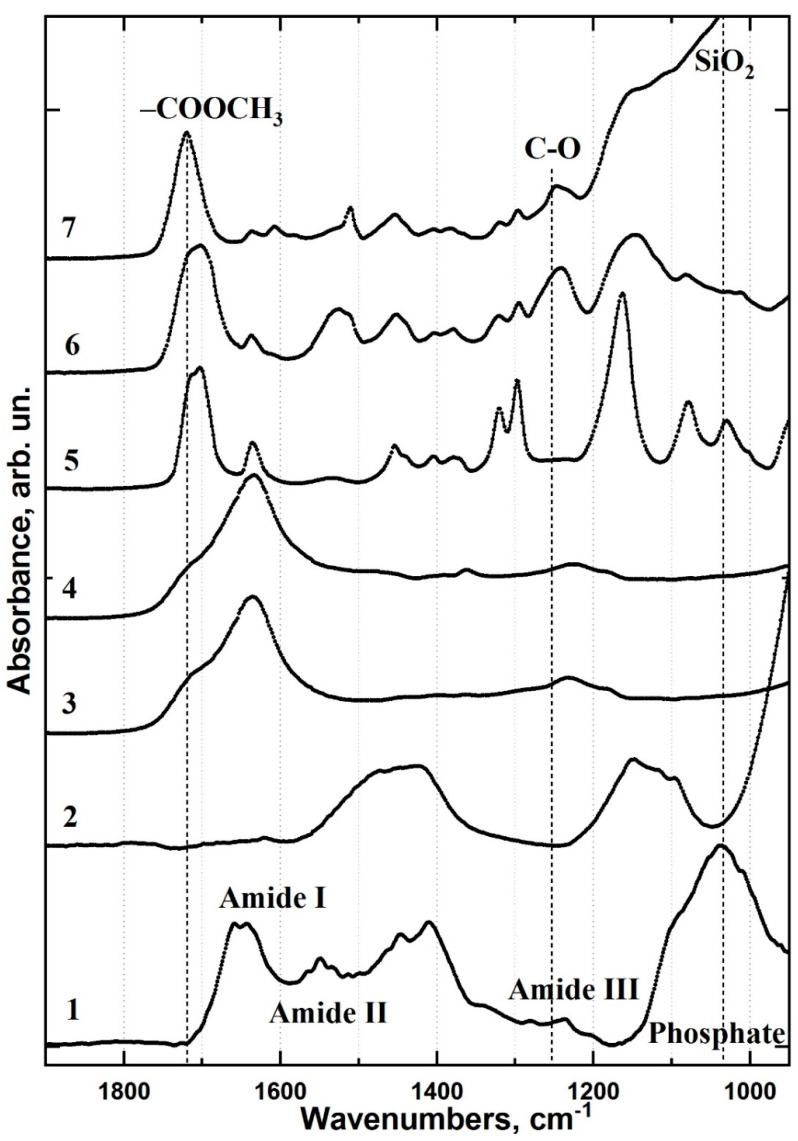
FTIR spectra of healthy dentine and the interface materials used for the type I and type II specimens: (1) healthy dentine; (2) Trioxident solution; (3) dentine conditioner; (4) modified dentine conditioner; (5) bioprimer; (6) Bis-GMA-based commercial adhesive; (7) Dyract XP light-curing dental compomer.

To observe chemical differentiation of the interface areas, we selected three primary vibrational modes corresponding to the interface substances identified by FTIR mapping [22]. The IR spectra of healthy dentine and the materials used for the type I and type II treatments were obtained (Figure 2).

**Figure 3:**
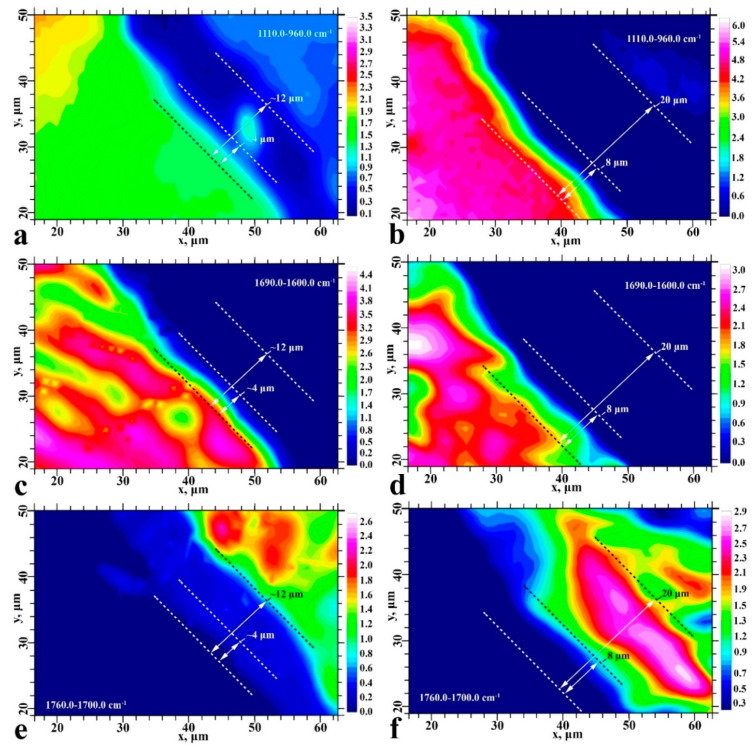
Chemical imaging maps for type I (**a**,**c**,**e**) and type II (**b**,**d**,**f**) specimens. Colour-coded univariate images of the mineral component of apatite dentine (**a**,**b**), amide I (**c**,**d**) and ester (-COOCH_3_) (**e**,**f**).

The first spectral band at 1110–960 cm^−1^ is associated with PO_4_ vibrations from the mineral component of dentine apatite and the SiO_2_ group in the Dyract XP compomer [28]. The second band between 1690–1600 cm^−1^ represents amide I (N-H, C=O, COO-) and corresponds to vibrations from proteins in dentine collagen and from the hybrid interface materials used in type II specimens [28,29,30]. The third spectral band at 1750–1700 cm^−1^ can be assigned to the vibrations of the ester group (-COOCH_3_) in the Bis-GMA adhesive and Dyract XP compomer [29]. FTIR maps were constructed for the three regions (1750–1700, 1690–1600 and 1110–960 cm^−1^) and describe the spatial distribution of the mineral (apatite) and organic (collagen) components, as well as the adhesive and dental material in the type I and type II interface areas (Figure 3).

The chemical imaging maps indicate homogeneous spatial distribution of mineral intensity in the healthy dentine area of both type I and II specimens (Figure 3a,b). The amide I band maps clearly show a specific spatial distribution of collagen and protein in the dentine zone due to the dentinal tubules located in this area (Figure 3c,d). The ester group (-COOCH_3_) FTIR maps for both type I and type II samples show non-homogeneous intensity distribution with characteristic zoning that delineates the border of the dental material and coincides with the border observed in the optical images (Figure 1 and Figure 3e,f). A detailed analysis of the interface areas of all three spectral regions suggests the presence of a transition layer between the healthy dentine zone and the adhesive/dental material zone, measuring ~12 μm wide in type I specimens and ~20 μm wide in type II specimens (Figure 3). This wider interfacial area potentially indicates interfacial interactions associated with the formation of a zone of demineralised and disorganised dentine and the graded penetration of the adhesive [22].

One-dimensional IR maps showing the interfacial distribution of apatite, collagen and adhesive/dental material are insufficient for describing integration processes owing to their limited ability to resolve spectral changes arising from minor chemical structure differences. Spectral bands used in chemical imaging often overlap with other vibrations. For example, the absorption bands of dentine phosphate groups overlap with the vibrations of aluminium silicates and silicon oxide found in dental material [29,31]. In addition, there are a number of overlapping bands within the 1718–1600 cm^−1^ region other than the amide I band [32]. This uncertainty in spectral assignments makes it impossible to analyse transition layers with subtle, graded changes in composition. However, this can be overcome using multidimensional clustering methods that permit efficient, systematic analysis of a large number of multicomponent IR spectra [33].

### 2.2. Cluster Analysis

**Figure 4:**
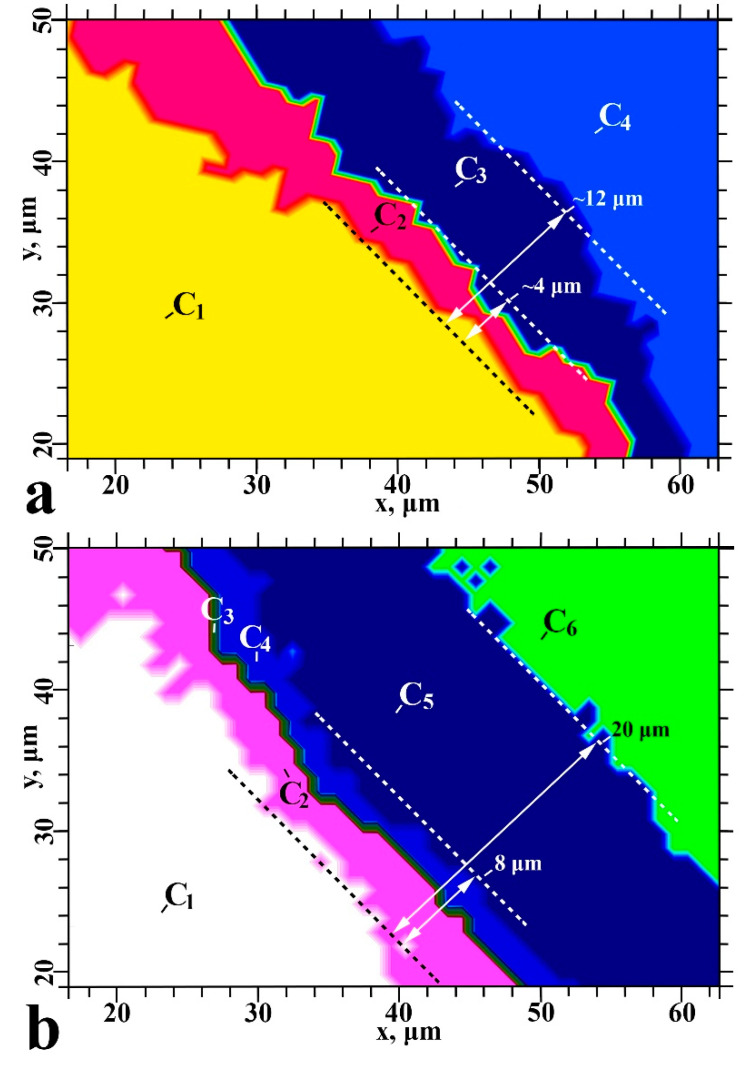
Cluster analysis for type I (**a**) and type II (**b**) samples. Colour-coded clusters (C_1_–C_4_ and C_1_–C_6_ for type I and II samples, respectively) were defined from the spectra using Ward’s method.

Cluster analysis groups the collected array of experimental spectral data into clusters, so that spectra within the same cluster have a clear affinity (maximum closeness based on the closeness function used during cluster analysis) and are significantly different from spectra in other clusters (Figure 4). The number of clusters for type I and type II samples was determined based on the heterogeneity dendrogram, accounting for technical data.

The healthy dentine/adhesive/dental material interface areas of type I and type II specimens are characterised by four (Figure 4a) and six (Figure 4b) clusters, respectively. The width of the type I and type II interface regions are noticeably different at ~12 and ~20 μm, respectively, which coincides with the chemical imaging. The lateral arrangement of the clusters allowed us to zone the characteristic areas of the type I and type II interfaces. Notably, the type II samples have more pronounced, straight boundaries between neighbouring clusters.

**Figure 5:**
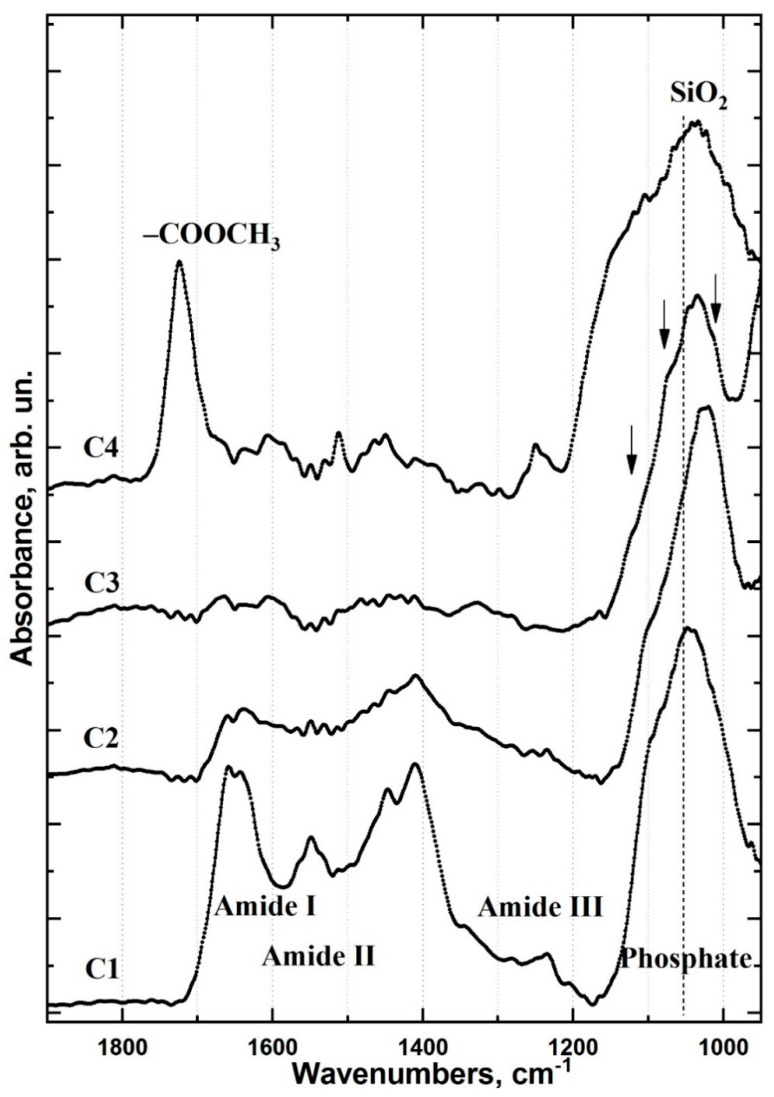
FTIR spectra of type I specimen clusters that are referred to colour-coded clusters C_1_–C_4_ for type I specimens in Figure 4a: (C_1_) FTIR spectrum of type I C_1_ cluster (healthy dentine); (C_2_) FTIR spectrum of type I C_2_ cluster (partially demineralised/disorganised dentine); (C_3_) FTIR spectrum of type I C_3_ cluster (laser-treated dentine); (C_4_) FTIR spectrum of type I C_4_ cluster (adhesive and dental material in the interface zone).

**Figure 6:**
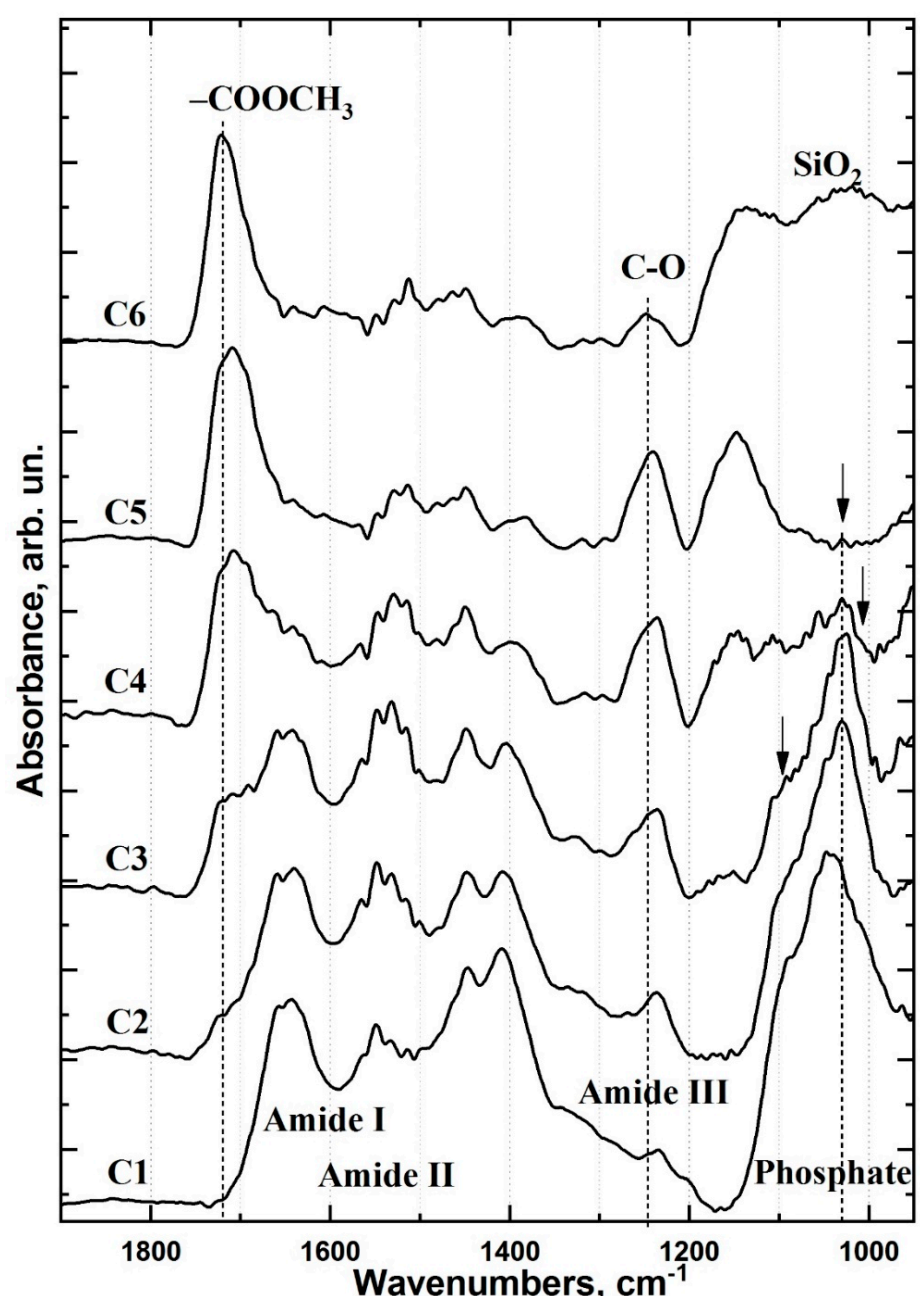
FTIR spectra of type II specimen clusters that are referred to colour-coded clusters C_1_–C_6_ for type II specimens in Figure 4b: (C_1_) FTIR spectrum of type II C_1_ cluster (healthy dentine); (C_2_) FTIR spectrum of type II C_2_ cluster (partially disorganised dentine); (C_3_) FTIR spectrum of type II C_3_ cluster (biointerface transition region); (C_4_) FTIR spectrum of type II C_4_ cluster (adhesive and dental material in the interface zone with partially disorganised dentin); (C_5_) FTIR spectrum of type II C_5_ cluster (nano-c-HAp–modified Bis-GMA adhesive); (C_6_) FTIR spectrum of type II C_6_ cluster (Dyract XP light-curing dental compomer).

To establish how the interface region and its chemical composition differ between the type I and type II samples, the averaged spectra of each cluster were extracted (Figure 5 and Figure 6, respectively). The reported FTIR spectra of healthy dentine, dental material, adhesive and the materials used for producing the type II specimen’s biomimetic hybrid layer were used as references for spectral data analysis.

**Figure 7:**
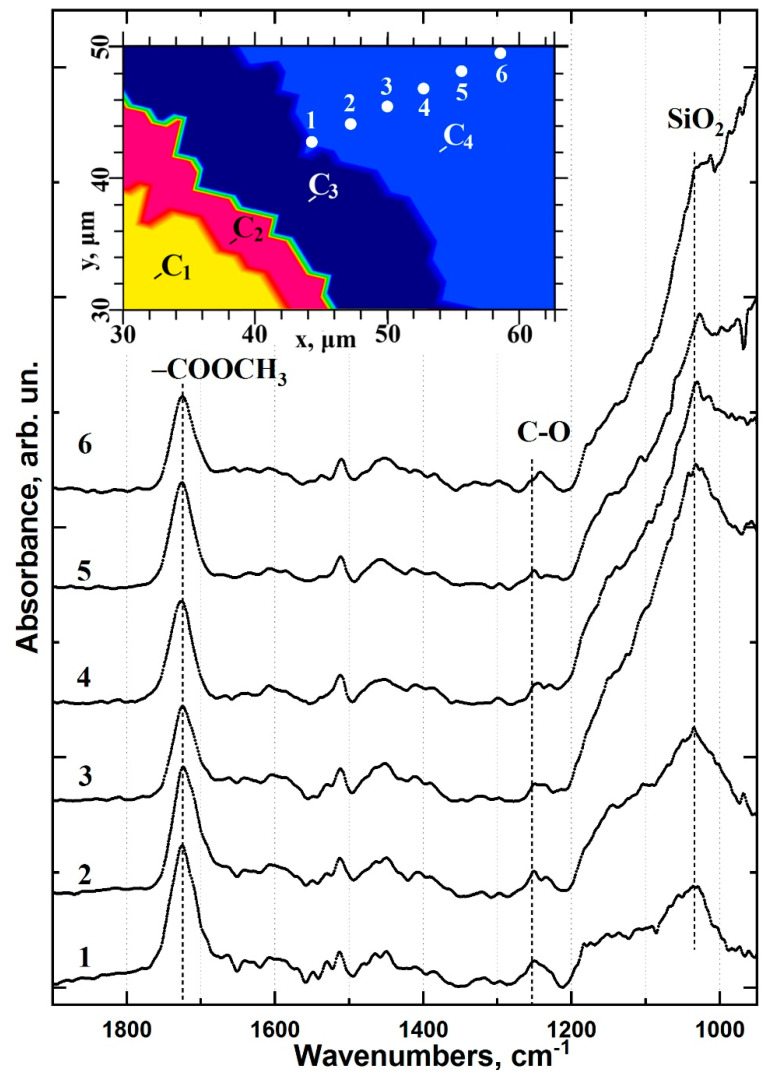
Differential chemical composition in the C_4_ (bioprimer and modified adhesive zone) cluster area perpendicular to the type I interface boundary based on FTIR data. (1, 2, 3, 4, 5, 6) FTIR spectra in points 1, 2, 3, 4, 5, 6 of C_4_ cluster, respectively (insert).

The C_1_ cluster in type I specimens is consistent with an area of healthy dentine (Figure 5). The C_2_ cluster represents an area of partially demineralised/disorganised dentine resulting from the acids in the dentine conditioner and laser irradiation (Figure 5). The spectral data for the C_3_ cluster indicates low-intensity residual oscillations related to organic components (collagen, proteins) along with an intense mineral (phosphate) band (Figure 5). This is characteristic of laser-treated dentine (LTD) tissue [34] consistent with the method of cavity formation during sample preparation. Laser irradiation is known to form a lubricated layer [34]. In this work, a dentine conditioner containing saturated and unsaturated acids was used to remove the LTD; however, it was still apparent in the spectra of the C_2_ and C_3_ clusters of the type I specimens. The C_4_ cluster shows evidence of adhesive and dental material in the interface zone (Figure 5). The differential chemical composition in this area indicates the presence of a thin layer of adhesive (a few microns) at the LTD boundary. Distal from the C_3_/C_4_ boundary, the contribution of the adhesive decreases while that of Dyract XP increases (Figure 7). Analysis of the spectral data and the characteristic features in the IR spectra of the adhesive and Dyract XP revealed that they mixed during the formation of the type I interface. The remnants of the LTD layer did not allow the adhesive to penetrate into the dentinal tubules, which was confirmed by the absence of characteristic bioprimer bands in the C_2_ and C_3_ clusters.

The outermost clusters in the type II specimen interface represent regions of healthy dentine (C_1_) and Dyract XP (C_6_) (Figure 6). The transition region between C_1_ and C_6_ contains four clusters. The C_2_ cluster, adjacent to the healthy dentine, is characterised by partially disorganised dentine (Figure 6). A comparison of the IR spectra of type I and II specimens’ C_2_ clusters suggests that the type II organic matrix is less altered than the corresponding area in type I specimens (Figure 5 and Figure 6). The additional Trioxident treatment and gentle etching with the organic acid-modified dentine conditioner performed during the creation of the type II biointerface more effectively removed the LTD components and opened the dentinal tubules. This was confirmed by the presence of all healthy dentine spectral features in the C_2_ IR spectrum, along with a change in the intensity and position of the amide and phosphate bands (Figure 6).

The type II C_3_ cluster has a narrow zone (~1–2 μm) in the biointerface transition region (Figure 6). The intensity of the υ1 and υ3 phosphate spectral components in the 1150–1100 cm^−1^ region of C_3_ changes, indicating a change in the phase composition of the dentine mineral component (Figure 6). The C_3_ area, located on the dentine surface boundary, displays a significantly different dentine apatite structure than that observed in C_2_, where partially disorganised dentine is located (Figure 6). The appearance of the C_3_ cluster in type II specimens results from the gentle etching of the dentine surface with dentine conditioner and the penetration of bioprimer into the dentine tissue. The former is evident from the band intensity change in the 1260–1240 cm^−1^ region and the latter is apparent from the increased mode intensity around 1720 cm^−1^, the stable 1640–1620 cm^−1^ position and low-intensity bands in the 1300–1180 cm^−1^ region (Figure 6).

The type II C_4_ cluster displays characteristic oscillations associated with the bioprimer and modified adhesive (Figure 6). Despite the similarities observed in the spectra of the pure components, their positions differ noticeably in the interface. The inclusion of both the bioprimer and modified adhesive in C_4_ is indicated by band broadening and increased vibrational intensities in the 1200–1100, 1280–1220 and 1750–1710 cm^−1^ regions (Figure 6). Notably, the C_4_ 1150–1100 cm^−1^ region contains two vibrational modes centred around 1125 and 1110 cm^−1^, which correlate with HPO_4_ [35] and phosphate-calcium complex vibrations and likely arise from the use of alkaline calcium treatments (Figure 6).

The nano-c-HAp–modified Bis-GMA adhesive used for the type II biointerface can be observed in C_5_, with the primary vibrational band intensities redistributed compared to the spectrum of the pure, unmodified adhesive (Figure 6). C_5_ also shows low-intensity features in the 1025 cm^−1^ region that are not present in the spectrum of the original adhesive. These are associated with the most intense mode of nano-c-HAp and appear weakly in the C_5_ spectra due to its low concentration in the modified adhesive.

The degree of conversion of adhesive material during polymerisation can be determined from FTIR data as the ratio of absorbance intensities before and after polymerisation: (C=C)/(C-C) [36]. The fraction of C=C and C-C bonds can be determined from the intensity of valence vibrations at ~1638 cm^−1^ and stretching vibrations of the aromatic ring at ~1607 cm^−1^, respectively. The proportion of non-polymerised bonds when using commercial Bis-GMA adhesive was 22.0 ± 1.4% (mean ± SD; *n* = 10), which agrees with previous reports for Bis-GMA/HEMA [36]. The proportion of non-polymerised bonds when using nano-c-HAp–modified Bis-GMA was 16.8 ± 1.7% (*n* = 10). A similar calculation was performed for modified adhesive in the C_5_ cluster of type II specimens and showed that the proportion of non-polymerised bonds in this region of the biomimetic interface was 18.9 ± 1.6%, similar to the modified adhesive alone.

## 3. Discussion

The stability and durability of the hybrid layer between the restorative material and dental hard tissue depend on the type of chemical bonding in the interfacial region and the interface quality [7,15]. Chemical imaging and HCA were used in this work to visualise and identify the transition layer regions of the hetero-interface. Comparative analysis showed that both the thickness and chemical composition of the hybrid layer differed significantly between type I and type II interfaces (Figure 3 and Figure 4).

The use of a three-component commercial bonding system (type I specimens) resulted in a minimal number of interface zones and did not generate a chemically and structurally homogeneous interface between the dentine, adhesive and dental composite. Scanning perpendicular to the type I interface revealed a thin layer of adhesive along the dentine interface (Figure 7). The intensity distribution maps of the PO_4_ group at 1110–960 cm^−1^ and the -COOCH_3_ ester at 1760–1700 cm^−1^ confirmed that the adhesive components did not penetrate into the dentine (Figure 3). This observation was consistent throughout the type I specimen hybrid layer boundary in the demineralised/disorganised dentine region, reflected in the shape of the C_2_ and C_3_ clusters, and agrees with previous findings (Figure 4) [22]. A layer of lubricated dentine created by laser ablation may impair interface formation in type I specimens. The use of laser light for cavity preparation in dentine has been shown to produce a disorganised LTD layer in the subsurface region containing amorphous calcium phosphate, which must be removed to achieve a quality interface [34]. Various etching primers have been employed to remove the lubricated layer and open the dentinal tubules [4,37]; however, active acids often lead to the formation of a thick layer of demineralised dentine [23] and the deterioration of interface characteristics [23]. The commercial bonding system employed for the type I specimens recommends the removal of the lubricated dentine layer using a single application of dentine conditioner containing low concentrations (≤12%) of saturated and unsaturated polyfunctional organic acids. This study found that a single application of conditioner did not qualitatively remove the LTD and bioprimer components did not penetrate the dentine, apparent in the absence of vibrational bands characteristic of the bioprimer from the C_2_, C_3_ and C_4_ spectra (Figure 5). As a result, no qualitative hybrid layer was formed in type I specimens.

The treatment of dentine with polar amino acid–modified dentine conditioner and alkaline calcium treatment (Trioxident solution) (type II specimens) resulted in less demineralised/disorganised dentine (cluster C_2_, Figure 4) than in type I specimens, which provides a higher quality interface. Subsequent application of a polar amino acid–modified primer promoted the opening of the dentinal tubules and penetration of the bioprimer components into the dentine, allowing the formation of a deeper transitional hybrid layer. The use of organic acids and Trioxident solution for the formation of the type II biointerface leads to an excess of calcium in the dentine surface layer (clusters C_3_, C_4_), which favours the binding of calcium to phosphate complexes (HPO_4_ and PO_4_) as observed in the FTIR spectra (Figure 6). The formation of stable dicalcium phosphate dihydrate and octacalcium phosphate can only occur on the surface of apatite nanocrystals but is reduced by repeated treatment with alkaline calcium and organic acids. This resulted in the redeposition of nano-c-HAp in the dentine surface layer upon formation of a non-apatite environment, which was observed as a maximum in the IR spectrum near 1107 cm^−1^, similar to previous work (Figure 6) [38].

The incorporation of remineralising or antibacterial agents [39] into adhesive systems is known to allow hybrid interface formation on polymer, collagen and resin, replacing the native dentine tissue at the dentine/adhesive interface [1]. This approach has already yielded positive results, producing stable bonds between adhesive and dentine. In addition, modifying Bis-GMA adhesive with nano-c-HAp, which is similar in morphology and structural organisation to natural apatite, increases the degree of conversion of the adhesive material during polymerisation.

The hybrid layer at the adhesive/dentine interface should ideally be a three-dimensional polymer/collagen network [36]. This type of interface formation between the natural tooth tissue and dental material is typical of existing commercial bonding systems. An amino acid–modified primer, alkaline calcium and nano-c-HAp-modified adhesive can be used to achieve a biomimetic hybrid layer at the dentine/dental material interface. FTIR chemometrics and HCA indicated that the biomimetic interface formed using this strategy (type II specimens) is chemically homogenous throughout the dentine and hybrid layer, ensuring optimal integration of the dental composite with the dentine. Hybrid interface engineering using biomimetic strategies is thus a promising new approach for restorative personalised nanodentistry.

## 4. Materials and Methods

### 4.1. Tooth Preparation and Treatment

#### 4.1.1. Tooth Samples

Ten (*n* = 10), healthy molars extracted from patients aged 18–25 due to orthodontic indications at the Burdenko Voronezh State Medical University dental clinic were used in this study. The patients were physically healthy based on outpatient records and had no unhealthy behaviours, including smoking. Teeth were collected in accordance with the relevant regulations. Informed consent was obtained from all participants and data collection and handling followed the Helsinki declaration. The study was approved by the Ethics Committee of Voronezh State University (Permission No. 003.012-2019, 12 December 2019). The extracted teeth were stored at 4 °C in individual vials containing 0.9% physiological solution and 0.002% sodium azide (Sigma-Aldrich: St. Louis, MO, USA).

#### 4.1.2. Treatment of Tooth Samples

The occlusal upper crown of the tooth was first cut perpendicular to the tooth axis using a low-speed, water-cooled diamond saw. A cylindrical cavity was then created in the dentine using a Dental laser PLUSER (Lambda S.p.A., Brendola, VI, Italy) Er:YAG pulsed laser (wavelength: 2940 nm, pulse duration: 75–500 µs, frequency: 10–50 Hz, maximum power: 8 W), and the cavity was washed and dried with compressed air. The cavity was then treated with a Trioxident (Vladmiva-Pharma Dental, Belgorod, Russia) solution (main component: calcium oxide) to obtain an alkaline pH (>11), which has been shown to activate the formation of functional molecular hydroxyapatite–amino acid bonds [40].

The prepared teeth were randomly divided into 2 equal treatment groups. Type I specimens (5 teeth) were treated with a bonding system comprised of a bisphenol-A-glycidyl methacrylate (Bis-GMA, Polysciences, Warrington, PA, USA, code 03344)-based commercial adhesive [11,25], Dyract XP (Dentsply Sirona CIS, Germany) light-curing dental compomer [41], dentine conditioner (Vladmiva-Pharma Dental, Belgorod, Russia) and bioprimer. Type II specimens were treated with Dyract XP and a bioprimer as well as modified dentine conditioner and Bis-GMA-based adhesive to create a hybrid biomimetic interface between the dentine and dental material.

For type I, the walls and base of the cavity were first treated with dentine conditioner for 60 s, rinsed with distilled water to remove residual lubricated dentine and dried with compressed air. A thin layer of bioprimer was then applied to the walls and bottom of the cavity according to the manufacturer’s instructions, and penetration of the bioprimer into the dentine tissue was activated using compressed air for 5 s. After 30 s, universal adhesive was applied to the cavity according to the manufacturer’s instructions and pre-cured under UV light for 10 s. Dyract XP was then applied and photopolymerised according to the manufacturer’s instructions.

For type II (5 teeth), the cavity was pretreated with Trioxident, treated with dentine conditioner for 60 s, rinsed with distilled water and dried with compressed air for 5 s. The Trioxident solution was reapplied, and the cavity was rinsed with distilled water and dried for 5 s. The cavity was then treated with the modified dentine conditioner for 60 s, rinsed with distilled water and dried. Trioxident solution was then applied to achieve an alkaline environment (pH > 11) on the surface layers of the pretreated dentine, and the cavity was dried with compressed air. The cavity was then treated with bioprimer for 60 s and compressed air was applied for 5 s to promote penetration into the dentine tissue. After 30 s, modified adhesive was applied to the cavity and pre-cured under UV light for 10 s. Dyract XP was then applied and photopolymerised according to the manufacturer’s instructions.

#### 4.1.3. Sectionalisation

Flat-parallel segments of the restored tooth samples were prepared for FTIR micro-mapping as previously described [42,43]. Tooth samples were separated using a low-speed, water-cooled diamond saw, and the resulting hard tissue layers were gently sanded and polished using diamond abrasive.

### 4.2. Materials

#### 4.2.1. Dentine Conditioner

The dentine conditioner was composed of a mixture of slightly concentrated (≤12%) saturated and unsaturated polyfunctional organic acids (maleic acid, polyacrylic acid, citric acid in distilled water. All chemical components were purchased from Sigma-Aldrich (St. Louis, MO, USA). Dentine conditioner removes “oiled dentine”, opens dentinal tubules and forms a functional bond between adhesive and dentine tissue.

#### 4.2.2. Modified Dentine Conditioner

The standard dentine conditioner was modified through the addition of lysine, arginine and hyaluronic acid (purchased from Sigma-Aldrich (St. Louis, MO, USA), which support the formation of a buffer layer and bonding to the dentine apatite. The amino acids were dissolved in an aqueous solution and mixed with the original dentine conditioner using a QSonica Q55 (Qsonica LLC, Newtown, CT, USA) sonicator.

#### 4.2.3. Bioprimer

The bioprimer contains compomer components (ethylene glycol methyl ester 30–85%, urethane dimethacrylate 1–15%, diglycidyl methacrylate hydrophilic monomer 1–15%) and a complex of polar amino acids (histidine 0.01–0.2%, lysine 0.05–0.4%, arginine 0.2–1.6% of the total primer weight), which support the synthesis of basic proteins. Bioprimer is used to introduce bonding components into the dentinal tubules and form a hybrid layer with the prepared dentine tissue.

#### 4.2.4. Modified Adhesive

Powdered nano-c-HAp was added to the Bis-GMA-based adhesive at a ratio of 1 mL:0.01 g adhesive: nano-c-HAp and mixed using a QSonica Q55 sonicator. The nano-c-HAp adhesive prevents stresses that can form during polymerisation and increases the hardness of the layers [44].

#### 4.2.5. Nanocrystalline Calcium Carbonate-Substituted Hydroxyapatite

Nano-c-HAp was obtained from bird eggshells via liquid-phase synthesis as previously described [45]. The morphological organisation of nano-c-HAp synthesised using this technique resembles that of human dental hard tissues, as it is formed by nanocrystals with an average size of 20 × 20 × 50 nm [46]. This characteristic is important for the formation of a biomimetic material capable of replacing natural biogenic HAp [47,48].

### 4.3. Synchrotron FTIR Microspectroscopy

Synchrotron FTIR microspectroscopy was performed on a Bruker VERTEX 80v spectrometer coupled with a HYPERION 3000 FTIR microscope and liquid nitrogen-cooled narrow-band mercury cadmium telluride detector (Bruker Optik GmbH, Ettlingen, Germany) at the Infrared Microspectroscopy beamline (Australian Synchrotron, Victoria, Australia). All FTIR spectra were recorded within a spectral range of 3800‒700 cm^−1^ using 4 cm^−1^ spectral resolution. Blackman-Harris 3-Term apodisation, Mertz phase correction and a zero-filling factor of 2 were used as default acquisition parameters in the OPUS v.7.5 software suite (Bruker Optik GmbH, Ettlingen, Germany).

Tooth slices were mounted on a thick polymer substrate using an epoxy adhesive. The top surfaces of the mounted samples were then polished with diamond paste. To avoid scattering artefacts commonly observed in reflectance spectra, the samples were analysed and imaged via macro attenuated total reflectance (ATR)-FTIR mapping using an in-house developed macro ATR-FTIR device equipped with a germanium (Ge) ATR crystal (250 μm diameter facet; *n*_Ge_ = 4.0), and a 20× IR objective (numerical aperture (NA) = 0.60; Bruker Optik GmbH, Ettlingen, Germany) [49,50]. The unique combination of the high refractive index of the Ge ATR crystal and the device’s high NA objective coupled to the synchrotron-IR beam allows surface characterisation of tooth slices at high resolution and without scattering artefacts.

The polymer substrate–mounted polished tooth slice was mounted onto an aluminium disc with double-sided polyimide (Kapton^®^) tape, which was placed onto the macro ATR-FTIR sample stage. The Ge ATR crystal was then brought to the focus of the synchrotron-IR beam, and a background spectrum was recorded at ambient conditions from 256 co-added scans. The tooth sample was then brought into contact with the Ge ATR crystal, and a low-resolution, overview, synchrotron macro ATR-FTIR chemical map was acquired to assess the area and the quality of the contact at 5 μm step intervals from 8 co-added scans. Subsequent synchrotron macro ATR-FTIR measurements were performed on areas of interest selected from the overview map using a step interval of 1 μm and 32 co-added scans.

### 4.4. Hierarchical Cluster Analysis

The 3D FTIR maps were evaluated using hierarchical cluster analysis (HCA), a multivariate statistical approach for classifying spectroscopic data. HCA permits the identification of regions within a sample structure based on their spectral response. Regions where the points display similar spectral responses demonstrate minimal intra-cluster spectral differences, while those with different spectral responses show maximal inter-cluster differences [23]. As the major vibrations characterising the investigated materials are within the 1800–950 cm^−1^ region of the IR spectrum, second derivative and vector normalisation were applied to this region to process raw spectral data for HCA. The spectra were smoothed over 17 points.

The optimal clustering algorithm was experimentally determined, using Euclidean distance as the measure between clusters. Ward’s method was used for clustering and construction of heterogeneity dendrograms. This method generates multiple partitions of the original image and considers all cluster (similar spectra) combinations using analysis of variance to assess the distance between clusters [51]. The number of clusters was determined based on technical data and the heterogeneity dendrogram. HCA was performed using OPUS v.7.5 software (Bruker Optik GmbH, Ettlingen, Germany).

## 5. Conclusions

This study revealed marked improvements in the formation of the dentine/adhesive/dental hybrid interface using a biomimetic approach. The use of a biomimetic strategy (application of an amino acid–modified primer, alkaline calcium and a nano-c-HAp–modified adhesive) in nanodentistry allowed the formation of a matrix that can be structurally integrated with natural dentine and dental composite. The biomimetic hybrid layer was characterised by homogeneous chemical composition and a higher degree of conversion of the adhesive during polymerisation, which should provide optimal integration of the dental composite with the dentine.

## 6. Ethics Declarations

All participants provided their written consent for participation. The Ethics Committee of Voronezh State University affirmed the performed examination (number of permission 003.012-2019). The examination was made in accordance with the approved principles.

All experiments and data collections were performed in accordance with relevant guidelines and regulations, including that all human participants provided informed consent and data collection and handling followed the Helsinki declaration.

## Data Availability

The data that support the findings of this study are available from the corresponding author upon reasonable request.
